# VEGF-A isoforms induce the expression of APLN in endothelial cells during human prenatal lung development

**DOI:** 10.3389/fcell.2025.1729884

**Published:** 2026-01-23

**Authors:** Antony Hoarau, Andrew Frauenpreis, Randa Belgacemi, Emma Loeffler, Osshaya Maalouf, Ian A. Glass, Denise Al Alam, Soula Danopoulos

**Affiliations:** 1 Department of Pediatrics, Lundquist Institute for Biomedical Innovation at Harbor-UCLA Medical Center, Torrance, CA, United States; 2 University of Washington School of Medicine, Seattle, WA, United States; 3 Department of Pediatrics, David Geffen School of Medicine at UCLA, Los Angeles, CA, United States

**Keywords:** angiogenesis, APLN, capillaries, human lung development, VEGF-A

## Abstract

**Introduction:**

Single-cell RNA-sequencing analyses have revealed the existence of two distinct capillary cell populations in the human lung: general capillary cells (CAP1) and alveolar capillary cells (CAP2). Studies in mouse have shown that the splicing of Vegf-a evolves during embryonic development, creating a temporal pattern of expression for different isoforms, which contributes to the formation of pulmonary capillaries. Moreover, it was demonstrated that murine Vegf-a188 isoform promotes the emergence of CAP2 *in vitro*. Human homologs of these VEGF-A isoforms exist; however, their role in this process remains elusive. This study investigates the role of VEGF-A and its isoforms in the differentiation of lung capillaries during human prenatal development.

**Methods:**

A cohort of human prenatal tissues, aged from the late pseudoglandular to early canalicular stages of development (10–20 weeks of gestation), was used to study the emergence of CAP2 markers *(TBX2, SOSTDC1, EDNRB, HPGD, APLN)* in correlation with the expression of the different VEGF-A isoforms *(VEGF-A121, VEGF-A145, VEGF-A165, VEGF-A189)*.

**Results:**

RT-qPCR analyses revealed a simultaneous expression of certain VEGF-A isoforms with several CAP2 markers, which peaked at around 18–20 weeks of gestation. Human prenatal lung explants were then treated with recombinant proteins of the different VEGF-A isoforms to study their impact on EC proliferation, as well as on the expression of CAP2 markers. While most of the isoforms did not impact EC proliferation, except for VEGF-A189 which downregulated it, almost all of them upregulated the expression of *APLN*, a major CAP2 marker. By using fluorescence *in situ* hybridization, we showed that this increase of expression was specific to the ECs. However, most of the isoforms induced a downregulation of *EDNRB* and *HPGD*. They also did not impact the expression of *SOSTDC1* and *TBX2*.

**Discussion:**

Our study shows that the different VEGF-A isoforms do not have the same effect on human lung capillary differentiation as those observed with their homologs in mice, highlighting the importance of studying this process in the human model. Moreover, while it demonstrated that VEGF-A isoforms can induce APLN expression in ECs, it also revealed that CAP2 differentiation is most likely a multifactorial process, not only involving VEGF-A.

## Graphical Abstract

**Figure:**
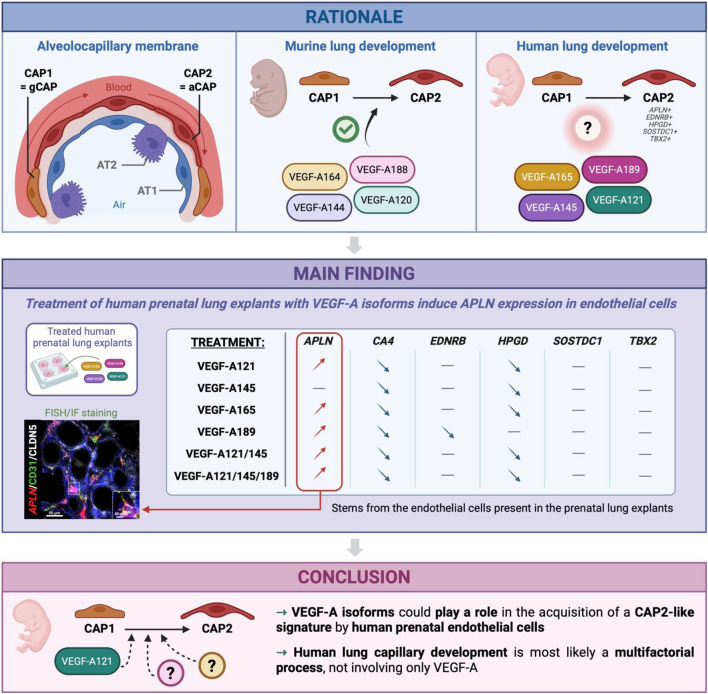

## 1 Introduction

Lung capillaries, in association with alveolar epithelial cells, comprise the functional gas exchange unit of the respiratory system. It was recently described that the endothelium of lung capillaries is composed of two distinct endothelial cell types: general capillary endothelial cells/CAP1 and alveolar capillary endothelial cells/aerocytes/CAP2 (Gillich et al., 2020). CAP1 are widely distributed and act as progenitors for CAP2 during homeostasis and tissue repair, whereas CAP2 cells are lung-specific and specialized for gas exchange and leukocyte trafficking (Gillich et al., 2020).

During embryonic development, CAP1 are primarily located in a vascular plexus. The transformation of this plexus, through the differentiation of CAP1 into CAP2, leads to the development of the alveolar capillary network. In mouse, CAP2 emerge at embryonic day E17.5 and are characterized by their extensively fenestrated structure (Gillich et al., 2020). Their number increase gradually during prenatal development but expand exponentially after birth, when they acquire a mature CAP2 transcriptional signature and phenotype (Gillich et al., 2020; Vila et al., 2020).

Several RNA sequencing studies have revealed distinct molecular signatures of CAP1 and CAP2 between mouse and human. Murine CAP1 are characterized by a high expression of *Plvap*, *Cd93*, *Ptprb*, or *Aplnr,* whereas human CAP1 are mainly defined by *APLNR*, *FCN3*, *EDN1*, *SLC6A4* or *VWF* (Gillich et al., 2020; Schupp et al., 2021; Cantu et al., 2023). These molecular differences are also found in CAP2. While murine CAP2 are molecularly defined by *Car4*, *Ednrb*, *Tbx2*, *Apln*, *Fibin;* human CAP2 are primarily characterized by *APLN, HPGD*, *EDNRB*, *SOSTDC1*, *ADIRF*, and S100A4. The bidirectional signaling between APLN (ligand) from CAP2 cells with *APLNR* (receptor) present on CAP1 cell surface is particularly noteworthy as it clearly demonstrates communication between the two populations and their role in maintaining pulmonary capillary homeostasis and function. Furthermore, although *Car4* is considered as a specific CAP2 marker in mouse, its human homolog *CA4* is considered a common EC marker (Gillich et al., 2020; Schupp et al., 2021; Cantu et al., 2023).

Vegf-a signaling has been identified as a critical pathway for the differentiation and maintenance of CAP2 cells mice. VEGF-A is a growth factor expressed by various cell types and involved in cell migration, proliferation and differentiation, resulting in neovascularization and angiogenesis (Shibuya and Claesson-Welsh, 2006; Tuder and Yun, 2008; Kumar and Ryan, 2004). Notably, the deletion of murine *Vegf-a* results in a depletion of CAP2 cells without affecting CAP1 number (Vila et al., 2020; Trimm and Red-Horse, 2022).

Murine *Vegf-a* possesses 8 exons and undergoes alternative splicing, giving rise to several isoforms (*Vegf-a120*, *Vegf-a144*, *Vegf-a164*, *Vegf-a188* and *Vegf-a205*). The most common *Vegf-a* isoform, expressed between E11.5 and E15.5 in the mouse lung, is *Vegf-a164,* followed by *Vegf-a188* and *Vegf-a120*, whereas *Vegf-a188* is more expressed than *Vegf-a164* in the adult mice lung (Healy et al., 2000). It has also been described that deletion of *Vegf-a164* and *-188* isoforms impairs lung microvascular development and delays airspace maturation, demonstrating the importance of these *VEGF-A* isoforms in normal lung development (Galambos et al., 2002). Moreover, treatment of mouse lung explants with Vegf-a188 increases the number of *Car4*-positive endothelial cells (mouse CAP2 cells), confirming its importance in murine CAP2 differentiation (Fidalgo et al., 2022). In humans, *VEGF-A* isoforms retain the same exon arrangement as in mice but have a different nomenclature associated with the addition of an amino acid (i.e., the human equivalent of murine Vegf-a164 is VEGF-A165) (Arcondéguy et al., 2013; Mamer et al., 2020).

While VEGF-A signaling is critical in the establishment and differentiation of murine CAP2, its role in the human developing lung capillary network remains elusive. The substantial differences in molecular signatures and functional roles between human and murine CAP2 raise questions about whether their differentiation relies on the same signaling pathways. In this study, we aim to investigate the role of VEGF-A and its isoforms in human prenatal lung capillary development and in CAP2 differentiation.

## 2 Materials and methods

### 2.1 Human prenatal tissues

The human prenatal lung tissues used in this study (Supplementary Table S1) were collected under IRB approval (The Lundquist Institute 18CR-32223-01) and provided to the lab by the University of Washington Birth Defects Research Laboratory. Specimens were de-identified with the only information collected being gestational age (GA) from 10 to 20 weeks, sex and the presence of any known genetic or structural abnormalities. Informed consent was provided for each sample used in this study.

### 2.2 Human prenatal lung explant culture

Explants were cut from the distal part of human prenatal lungs at 250 µm using the McIlwain tissue chopper. Characteristics of the tissues used to obtain the explants are summarized in Table 1. Lung explants were cultured on Whatman nucleopore polycarbonate membranes (8 μm pore size, cat number #10417501, Cytiva) in Dulbecco’s Modified Eagle Medium/Nutriment Mixture F-12 (DMEM-F12, cat#11-320-082, Gibco) supplemented with 1% of Fetal Bovine Serum (FBS, cat#10082147, Gibco) and 1% Penicillin-Streptomycin (10,000 U/mL, cat#15-140-122, Gibco), and incubated at 37 °C with 5% CO_2_. The human lung explants were treated with recombinant human VEGF-A (rhVEGF-A) isoforms either individually (VEGF-A121, VEGF-A145, VEGF-A165, VEGF-A189) or in defined combinations (VEGF-A121 + VEGF-A145, and VEGF-A121 + VEGF-A145 + VEGF-A189), with untreated explants serving as the negative control. Each treatment was refreshed every 24 h over a 48-h culture period. The rhVEGF-A isoforms come from R&D system (VEGF-A121: cat#4644-VS-010, VEGF-A145: cat#7626-VE-010, VEGF-A165: cat#293-VE-010, VEGF-A189: cat#8147-VE-025). Considering the supplier information about the median effective dose, we selected 30 ng/mL, 20 ng/mL, and 80 ng/mL, respectively, for VEGF-A121, VEGF-A165, and VEGF-A145/VEGF-A189. Explants were subsequently collected for gene expression analysis or immunofluorescent staining. For each biological sample, we collected enough explants to complete the full experimental design: 14 explants per sample. These were divided into two sets for each of the seven treatment conditions: one set designated for RNA analysis and the other for histology and immunofluorescence.

**TABLE 1:** Characteristics of the human prenatal lung tissues used to obtain explants. None of the tissues presented with genetic or structural abnormalities. GA: gestational age.

Sample Identifier	Sex	GA
H29501	M	16.3
H29316	F	16.4
H29412	F	16.4
H29419	F	16.4
H29682	F	17
H29342	M	17.4
H29324	M	17.7
H29410	F	17.7
H29645	M	17.7
H29647	M	17.7
H29284	M	19.1
H29321	F	19.3
H29298	M	20.9
H29650	M	21.6

### 2.3 RNA extraction and RT-qPCR analysis

RNA from native human prenatal lungs ranging from the late pseudoglandular (10–15 weeks of GA, n = 24) to the early canalicular (16–20 weeks of GA, n = 28) stages of development, as well as from treated lung explants (n = 14), was extracted using easy-spin^TM^ Total RNA Extraction kit from iNtRON Biotechnology (cat#17221) based on the manufacturer’s protocol. cDNA synthesis was performed using the Tetro cDNA Synthesis Kit (Meridian Bioscience, BIO-65043). Gene expression analysis was performed in triplicate for each sample, using human-specific TaqMan gene expression assays (Table 2) and the TaqMan Fast Advanced Master Mix (Applied Biosystems, cat#4444557). For the different *VEGF-A* isoforms, we designed and validated primers, and probes were custom-made by ThermoFisher (Table 2). Product amplification was performed using the QuantStudio 3 Real-Time PCR System (Applied Biosystems). Relative gene expression was established using GAPDH as the housekeeping gene.

**TABLE 2:** Taqman assays and custom-made primers and probes used for RT-qPCR**.**

Target gene	Taqman assay or custom sequences
*Universal capillary marker*	
*CA4*	Hs00426343_m1
*CAP marker primer*	
*APLN*	Hs00175572_m1
*EDNRB*	Hs00240747_m1
*HPGD*	Hs00960586_m1
*SOSTDC1*	Hs00383602_m1
*TBX2*	Hs00911929_m1
*VEGF-A*	
*VEGF-Atot*	Hs00900055_m1
*VEGF-A121*	Forward: 5′-AAT​GTG​AAT​GCA​GAC​CAA​AGA​AAG-3′
	Reverse: 5′-ACC​CTG​AGG​GAG​GCT​CCT​T-3′
	Probe: 5′-6FAM-AGACAAGAAAAATGTGACAAGC-MGBNFQ-3′
*VEGF-A145*	Forward: 5′-CGC​AAG​AAA​TCC​CGG​TAT​AAG​T-3′
	Reverse: 5′-ACC​CTG​AGG​GAG​GCT​CCT​T-3′
	Probe: 5′-6FAM-CTGGAGCGTTTGTGACAAGC-MGBNFQ-3′
*VEGF-A165*	Forward: 5′-GTG​AAT​GCA​GAC​CAA​AGA​AAG​ATA​GA-3′
	Reverse: 5′-CCT​TGC​AAC​GCG​AGT​CTG​T-3′
	Probe: 5′-6FAM-CAAGACAAGAAAATCCCTGTGG-MGBNFQ-3′
*VEGF-A189*	Forward: 5′-CGC​AAG​AAA​TCC​CGG​TAT​AAG​TC-3′
	Reverse: 5′-CGC​GAG​TCT​GTG​TTT​TTG​CA-3′
	Probe: 5′-6FAM-CTGGAGCGTTCCCTGTG-MGBNFQ-3′
*Housekeeping gene*	
*GAPDH*	Hs02786624_g1

### 2.4 Explants embedding and sectioning

Cultured explants were fixed in 4% paraformaldehyde for 30 min at room temperature. Tissues were then dehydrated in an increasing series of ethanol concentrations, cleared in xylene, and embedded in paraffin. For ulterior analyses, formalin-fixed paraffin-embedded (FFPE) prenatal lung explants were cut in 5 μm sections using a microtome.

### 2.5 Hematoxylin and eosin staining

Sections were deparaffinized with xylene and rehydrated by a decreasing series of ethanol concentrations as previously described (Danopoulos et al., 2016). The sections were stained with Hematoxylin+ (Fisher Healthcare^TM^, 22-220-100) for 5 min, rinsed with distilled water for 5 min then stained with Eosin (Fisher Healthcare^TM^, 22-220-104) for 5 min. Tissues were then dehydrated by gradient ethanol solutions and cleared with xylene. The sections were assembled with DPX mounting medium (BDH, 360294H) and Corning cover glass (Corning, 2980-225).

### 2.6 Immunofluorescence (IF) staining

Lung explants were processed for immunofluorescent staining as previously described (Danopoulos et al., 2016). Briefly, after rehydrating, sections were blocked in 3% Bovine Serum Albumin, 10% normal goat serum, 0.1% Triton-X100 in TBS for 2 h. They were then incubated with the appropriate primary antibodies overnight at 4 °C in a humid chamber (Table 3). The next day, the slides were washed in TBST and incubated with appropriate secondary antibodies supplemented with DAPI for 1 h at RT. Slides were then mounted under a coverslip with Prolong^TM^ Gold Antifade Mountant (Invitrogen, P36934).

**TABLE 3:** Antibodies and RNAscope probe used for fluorescent immunostaining and RNA *in situ* hybridization**.**

Primary antibodies	Sources	Identifier	Dilution
CD31	Thermo Fisher	MA5-13188	1/100
Claudin-5	LSBio	LS-C352946	1/200
Ki67	Epredia	RM-9106-S1	1/200
DAPI	Abcam	AB228549	1/1000
RNAscope assay			
*APLN*-C3	ACDB	44–9971-C3	1/50
Secondary antibodies			
AlexaFluor 647	Jackson Immuno Research	115-606-104	1/500
AlexaFluor 488	Jackson Immuno Research	115-546-144	1/500
Opal 570	Akoya BioSciences	OP-001003	1/500
Cy3	Jackson Immuno Research	115-166-146	1/500

### 2.7 RNA fluorescent *in situ* hybridization coupled with IF

Fluorescent *In Situ* Hybridization (FISH) was conducted on 5 μm lung explant sections as previously described (Danopoulos et al., 2020), using the Advanced Cell Diagnostics RNAscope Fluorescent Multiplex Assay (Cat# 323110) per the manufacturer’s instructions. The probes were obtained from ACDBio, the signal was revealed with the Opal 570 (Table 3). Following FISH protocol, combinatorial IF staining was performed as previously described (Danopoulos et al., 2023).

### 2.8 Imaging and quantification

Ten pictures were captured at x40 objective on the Leica Thunder Imager DMi8 and quantified using HALO® Image Analysis Platform (version 3.6.4134, Highplex FL and FISH IF modules, Indica Labs, Inc; Albuquerque, NM).

### 2.9 Statistical analyses

Statistical analyses were performed using GraphPad Prism (GraphPad Software Inc., La Jolla, CA, United States). Data normality was assessed using the Shapiro–Wilk test. If the assumptions for parametric testing were met, comparisons between two groups were performed using a paired or unpaired *t*-test, as appropriate. For comparisons involving multiple groups relative to the untreated control, a repeated-measures one-way ANOVA was used, with Dunnett’s multiple comparisons test applied to adjust *P* values. If the assumptions for parametric testing were not met, a Wilcoxon matched-pairs test was used for two-group comparisons, and a Friedman test was applied for repeated-measures multiple-group comparisons, with Dunn’s multiple comparisons test used for adjustment. Statistical significance was defined as *p* ≤ 0.05.

A graphical of the experimental design of each step of the methodology described above is represented in Supplementary Figure S1.

## 3 Results

### 3.1 Expression of CAP2 cell markers and VEGF-A121, -145, −189 start peaking simultaneously during human lung development

**FIGURE 1:**
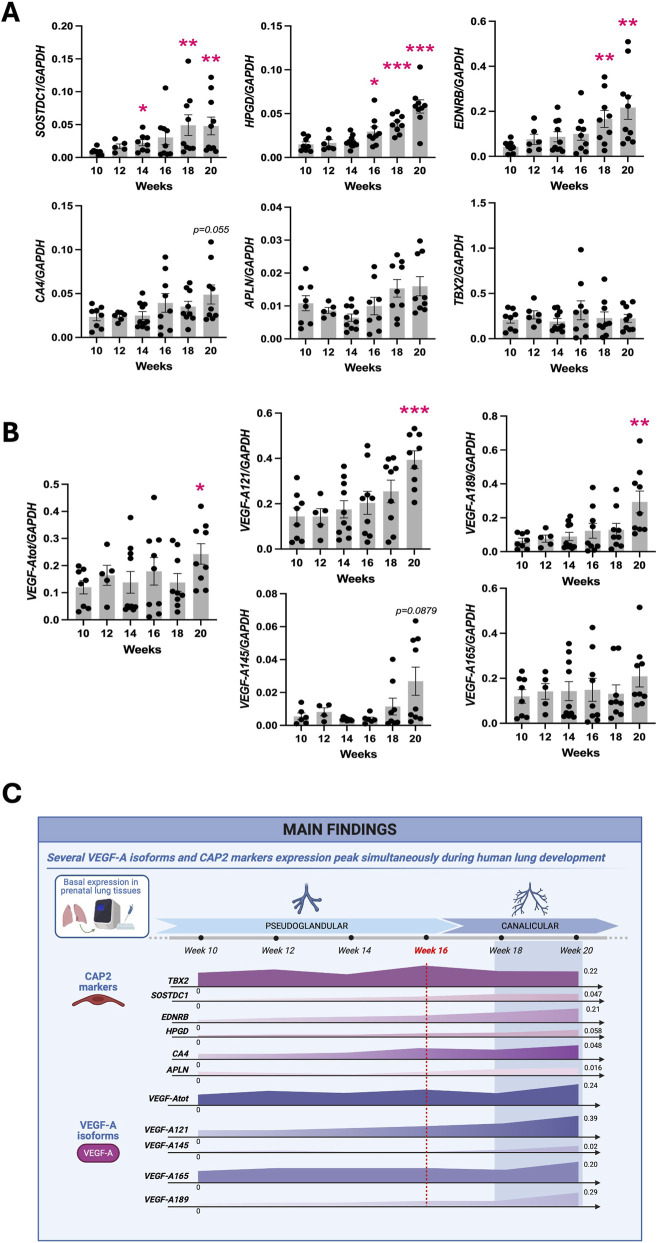
Expression of CAP markers and VEGF-A isoforms during human prenatal lung development. RT-qPCR analysis of the expression of universal capillary marker CA4 and CAP2 specific markers (*SOSTDC1*, *HPGD, EDNRB, CA4, APLN,* and *TBX2*) **(A)** and of total *VEGF-A* (*VEGF-Atot*) as well as each *VEGF-A* isoform (*VEGF-A121*, *VEGF-A145*, *VEGF-A165*, *VEGF-A189*) **(B)** in native human prenatal lungs aged from the late pseudoglandular to the early canalicular stages of development (week 10–20 of GA). Results are shown as a dot plot with mean ± SEM, n = 8–10, *p < 0.05, **p < 0.01, ***p < 0.001. **(C)** Visual summary of the *VEGF-A* isoforms and CAP2 markers expression during human prenatal lung development. Image created by Biorender.com.

The emergence of CAP2 in the human lung remains poorly described. Several groups have previously reported that CAP2 cells appear at around 18–21 weeks of gestation in the developing human lung (Gillich et al., 2020; He et al., 2022; Zepp et al., 2021; Bhattacharya et al., 2025). However, given the limitation of these datasets from single-cell RNA-sequencing (scRNA-seq), we explored this further. Using RT-qPCR, gene expression for CAP2 markers (*SOSTDC1, HPGD, EDNRB, APLN and TBX2)* were analyzed in native prenatal lung tissues aged from 10 to 20 weeks of GA, corresponding to the transition from the late pseudoglandular to early canalicular stages of lung development (Figure 1). Additionally, the expression level of *CA4* was also investigated, given its role as a CAP2 marker within the mouse lung, although it has been demonstrated to be a ubiquitous capillary marker within the human lung (Fleming et al., 1993). Interestingly, we noted that *SOSTDC1* increased significantly in two phases: first at 14 weeks (week 14: 0.01925 ± 0.004233, n = 10 vs. week 10: 0.0089 ± 0.001728, n = 8, *p =* 0.0168), and then again at 18 weeks (week 18: 0.04914 ± 0.01575, n = 9, *p* = 0.0055). *HPGD* expression started increasing significantly at 16 weeks (week 16: 0.02954 ± 0.005425, n = 9 vs. week 10: 0.01505 ± 0.002786, n = 8, *p* = 0.0372), whereas *EDNRB* demonstrated significantly increased expression starting at 18 weeks (week 18: 0.1661 ± 0.03757, n = 9 vs. week 10: 0.04244 ± 0.009032, n = 8, *p* = 0.0085). Moreover, we observed an upward trend in the expression of *CA4* at week 20 (week 20: 0.04885 ± 0.01083, n = 8 vs. week 10: 0.02343 ± 0.004332, n = 8, *p* = 0.055). In contrast, *APLN* and *TBX2* expression remained constant throughout the gestational ages.

Having established the expression of several CAP2 markers throughout lung development, we next investigated the potential implication of VEGF-A in CAP2 differentiation. Therefore, we analyzed the expression of *VEGF-A* in the native human prenatal lung samples using RT-qPCR (Figure 1B) and noted significantly increased expression of total *VEGF-A* (*VEGF-Atot)* at 20 weeks of GA as compared to 10 weeks (week 20: 0.2426 ± 0.03788, n = 9 vs. week 10: 0.1202 ± 0.02455, n = 8, *p* = 0.0187). To better evaluate the implication of each isoform, we designed and validated primers and probes for *VEGF-A121*, -*145*, -*165 and* -*189*. We observed an increase of *VEGF-A121* expression throughout the weeks, which started becoming significant at 20 weeks (week 20: 0.3943 ± 0.03885, n = 9 vs. week 10: 0.1440 ± 0.03749, n = 8, *p* = 0.0003). Similarly, at 20 weeks of GA, we noted significantly increased expression of *VEGF-A189* (week 20: 0.2935 ± 0.06408, n = 9 vs. week 10: 0.06349 ± 0.01541, n = 8, *p* = 0.0049) as well as an upward trend for *VEGF-A145* (week 20: 0.02684 ± 0.008570, n = 9 vs. week 10: 0.005533 ± 0.002014, n = 6, *p* = 0.0879). However, *VEGF-A165* expression did not change over time.

Altogether, these results indicate a simultaneous increase of expression of *VEGF-A121, -145,* and -*189* isoforms with several CAP2 markers (*SOSTDC1, EDNRB, HPGD*), peaking at around 18–20 weeks of GA (Figure 1C). Therefore, all subsequent experiments are performed using lung tissues at or older than 16 weeks of gestation.

### 3.2 VEGF-A189 affects endothelial cell proliferation

**FIGURE 2:**
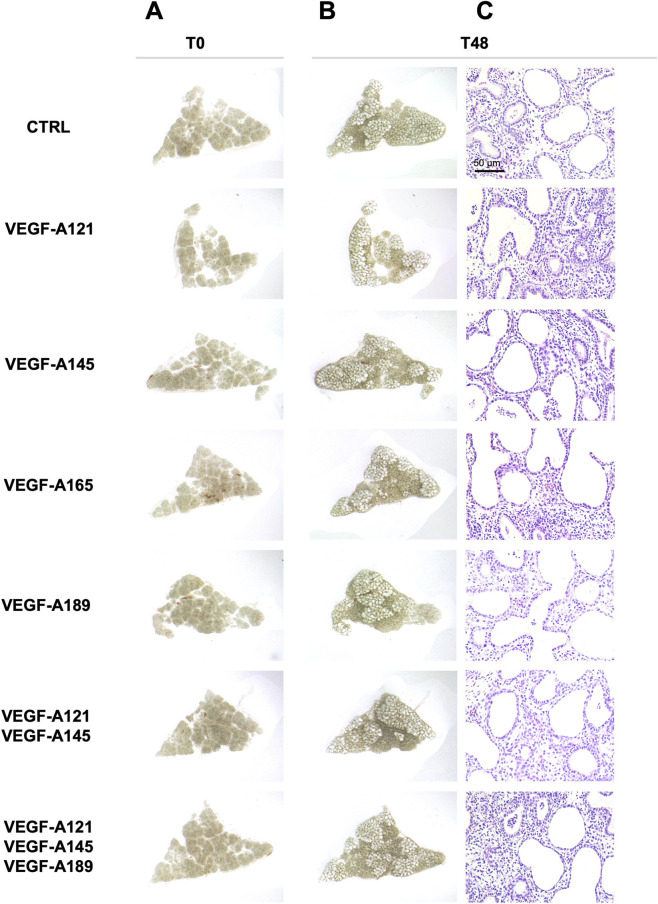
Effect of VEGF-A isoforms on human prenatal lung explant morphology. Representative pictures of the human prenatal lung explant cultures, either untreated or treated with the different VEGF-A isoforms alone or in combinations (VEGF-A121, VEGF-A145, VEGF-A165, VEGF-A189, VEGF-A121-145, and VEGF-A121-145–189) for 48 h. Photos were taken at time 0 (left, **(A)** and at 48 h (right, **(B)**. At 48 h, hematoxylin-eosin staining was performed to evaluate the histology of the explants **(C)**. Scale bar indicated by 50 µm.

Given the simultaneous expression of several VEGF-A isoforms with some CAP2 markers in the human prenatal lung tissues, it is possible that the different VEGF-A isoforms could be involved in CAP2 differentiation during human lung development. To verify this hypothesis, we treated human prenatal lung explants (starting at 16 weeks of GA) with exogenous recombinant human VEGF-A isoforms for 48 h (Figure 2). The different VEGF-A isoforms were either used alone or in combinations (VEGF-A121, -145 and/or −189) to explore whether a cumulative effect of several isoforms could be observed. We aimed to explore whether the different VEGF-A isoform treatments altered the normal growth of the lung explants. Explant morphology was observed at baseline (time 0; Figure 2A) and after 48 h of treatment (Figure 2B). No difference was determined in the overall aspect of the treated explants compared to the non-treated explants after 48 h (Figure 2B). Moreover, hematoxylin-eosin staining of the prenatal lung explants treated with VEGF-A isoforms displayed no visual histological difference when compared to the non-treated explants (Figure 2C).

**FIGURE 3:**
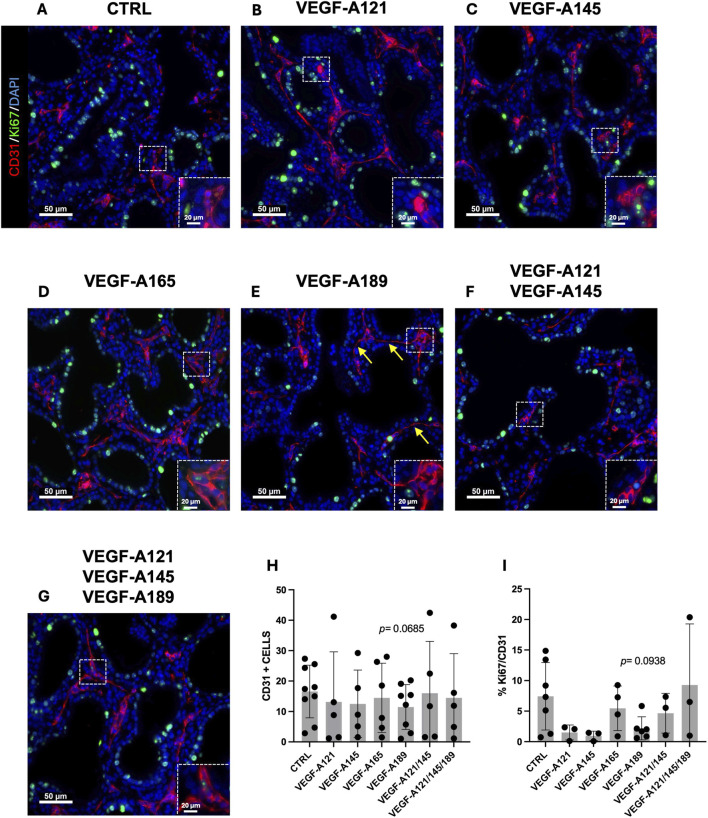
Impact of VEGF-A isoforms on endothelial cell proliferation in human prenatal lung explants. **(A–G)** Representative pictures of human prenatal lung explants, aged from the late pseudoglandular to the early canalicular stage (16–21 weeks of GA), untreated **(A)** or treated with VEGF-A121 **(B)**, VEGF-A145 **(C)**, VEGF-A165 **(D)**, VEGF-A189 **(E)**, VEGF-A121-145 **(F)** or VEGF-A121-145-189 **(G)** for 48 h, stained for CD31 (red) and Ki67 (green) by IF. Nuclei were counterstained with DAPI (blue). **(H,I)** Associated quantification of the number of CD31-positive cells **(H)** and the percentage of Ki67-CD31double-positive cells **(I)**. Results are shown as a dot plot with mean ± SEM, n = 3–9. Scale bar indicated by 50 µm.

We next aimed to investigate cellular-level alterations by examining how treatment influenced capillary proliferation. This was of interest given that CAP2 are considered to be highly differentiated and specialized cells, with limited to no proliferative capacity, whereas CAP1 are more associated as being the proliferative progenitor cells (Wong et al., 2024). We therefore performed a CD31 (PECAM-1) and Ki67 IF staining on all treatment conditions (Figures 3A–G) to explore the impact of the different VEGF-A isoforms on the number (Figure 3H) and proliferative capacity of the ECs (Figure 3I). Our data showed no difference in the number of CD31-positive cells when the explants were treated with VEGF-A121 (Figure 3B), VEGF-A145 (Figure 3C), VEGF-A165 (Figure 3D), or combined treatments VEGF-A121-145-189 (Figure 3G). However, a trend towards a reduced number of CD31-positive cells was observed in VEGF-A189 treated explants (11.48 ± 2.608, n = 6, *p* = 0.0685) (Figures 3E,H). Furthermore, VEGF-A189 was the only treatment that trended towards altering the proportion of proliferating ECs, displaying a decrease in the percentage of Ki67/CD31 double-positive cells compared to the control (VEGF-A189: 2.196 ± 0.7729, n = 6, *p* = 0.0938) (Figure 3I). This treatment also resulted in the ECs presenting with a more elongated and flattened morphology, as indicated by the yellow arrows in Figure 3E. The only other treatment that trended towards decreased EC proliferation was VEGF-A145 (VEGF-A145: 0.9595 ± 0.4462, n = 3, *p* = 0.1867).

### 3.3 VEGF-A isoforms are implicated in the CAP2-like cell signature

**FIGURE 4:**
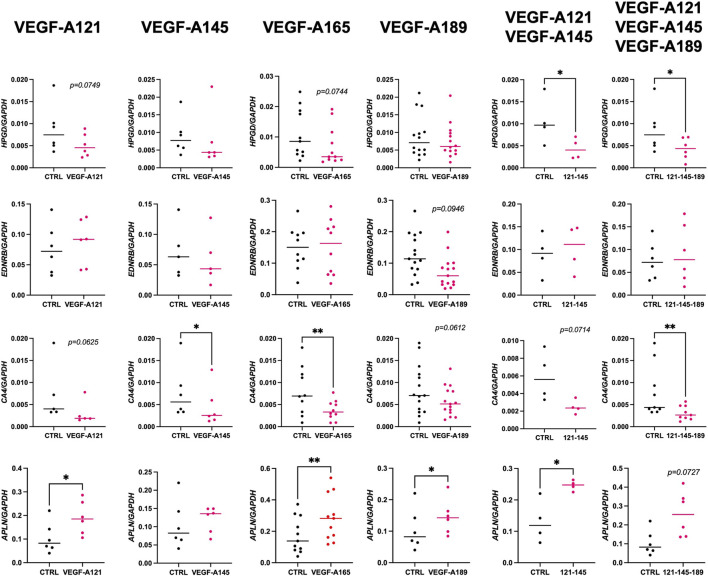
Impact of VEGF-A isoforms on the expression of CAP2 markers in human prenatal lung explants. RT-qPCR analysis of the expression of CAP2 markers (*HPGD*, *EDNRB*, *CA4,* and *APLN*) in human prenatal lung explants aged from the late pseudoglandular to the early canalicular stage (16–21 weeks of GA), untreated or treated with the different VEGF-A isoforms alone or in combinations (VEGF-A121, VEGF-A145, VEGF-A165, VEGF-A189, VEGF-A121-145, and VEGF-A121-145–189) for 48 h. Results are shown as a dot plot with mean ± SEM, n = 4–15, *p < 0.05, **p < 0.01.

Although there were no stark morphological differences within the endothelium of the VEGF-A treated explants, we want to understand if perhaps endothelial cell signature was being altered, suggesting a shift towards the establishment of CAP2 cells. Therefore, we next examined the expression of several CAP2 markers (*EDNRB, HPGD, CA4, and APLN* in Figure 4; *SOSTDC1* and *TBX2* in Supplementary Figure S2) on the different VEGF-A treated explants from 16 to 21 weeks of GA.

Overall, *SOSTDC1, HPGD*, and *CA4* expression levels were much lower than those of *APLN*, *EDNRB*, and *TBX2*, suggesting their role may not be as critical at this stage of development. None of the VEGF-A isoforms seemed to affect *SOSTDC1* and *TBX2* expression (Supplementary Figure S2). Alternatively, our data showed that VEGF-A isoforms may affect *HPGD, EDNRB, CA4, and APLN* expression. Treatments with VEGF-A121 and -165 isoforms alone demonstrated a downward trend in *HPGD* expression (VEGF-A121: 0.005114 ± 0.001072, n = 6, *p* = 0.0749; VEGF-A165: 0.007025 ± 0.001914, n = 11, *p* = 0.0744; VEGF-A145: 0.007554 ± 0.003142, n = 6, *p* = 0.4375). Combinations of VEGF-A121-145 and VEGF-A121-145-189 even significantly decreased *HPGD* expression (VEGF-A121-145: 0.004350 ± 0.001187, n = 4, *p* = 0.0393; VEGF-A121-145-189: 0.004304 ± 0.001005, n = 8, *p* = 0.0289). In contrast, VEGF-A189 induced no difference in *HPGD* expression (VEGF-A189: 0.007545 ± 0.001292, n = 14, *p* = 0.6698) but the only VEGF-A isoform to alter *EDNRB* expression was VEGF-A189, resulting in a decrease (0.07211 ± 0.01306, n = 15, *p* = 0.0946). Furthermore, VEGF-A121, -189, and 121-145 treatments induced a downward trend in *CA4* expression while VEGF-A145, -165 and 121-145-165 significantly decreased *CA4* expression (VEGF-A121: 0.003074 ± 0.001193, n = 5, *p* = 0.0625; VEGF-A145: 0.12 ± 0.001821, n = 6, *p* = 0.0312; VEGF-A165: 0.003730 ± 0.0006941, n = 10, *p* = 0.0045; VEGF-A189: 0.005723 ± 0.00085, n = 15, *p* = 0.0612; VEGF-A121-145: 0.002471 ± 0.0003921, n = 4, *p* = 0.0714; VEGF-A121-145-189: 0.003136 ± 0.0005311, n = 9, *p* = 0.0039). However, and most interestingly, all VEGF-A isoforms (except for VEGF-A145) induced a strong upward trend or even significantly increased *APLN* expression (VEGF-A121: 0.1906 ± 0.02878, n = 6, *p* = 0.0153; VEGF-A145: 0.1204 ± 0.01438, n = 6, *p* = 04,813; VEGF-A165: 0.2862 ± 0.04385, n = 11, *p* = 0.0011; VEGF-A189: 0.1454 ± 0.02257, n = 6, *p* = 0.0215; VEGF-A121-145: 0.2460 ± 0.008221, n = 4, *p* = 0.0352; VEGF-A121-145-189: 0.2575 ± 0.04870, n = 6, *p* = 0.0727).

### 3.4 VEGF-A isoforms induce APLN expression in endothelial cells

**FIGURE 5:**
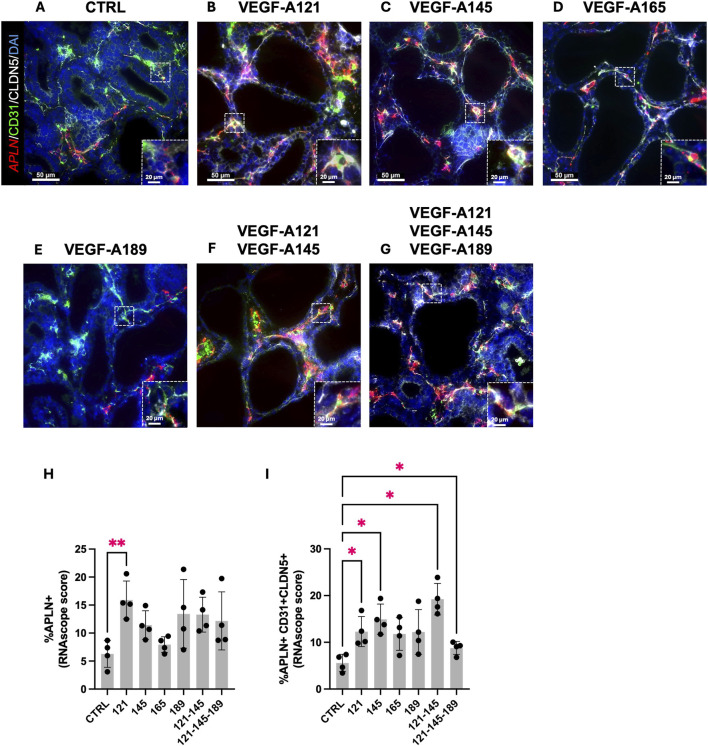
VEGF-A isoforms induce APLN expression in endothelial cells. **(A–G)** Representative pictures of human prenatal lung explants, aged from the late pseudoglandular to the early canalicular stage (16–21 weeks of GA), untreated **(A)** or treated with VEGF-A121 **(B)**, VEGF-A145 **(C)**, VEGF-A165 **(D)**, VEGF-A189 **(E)**, VEGF-A121-145 **(F)** or VEGF-A121-145-189 **(G)** for 48 h, stained for CD31 (green) and Claudin-5 (white) by IF, and for *APLN* (red) by FISH. Nuclei were counterstained with DAPI (blue). **(H,I)** Associated quantification of the percentage of *APLN* + cells **(H)** and the percentage of *APLN*+ CD31^+^ CLDN5+ cells **(I)**. Results are shown as a dot plot with mean ± SEM, n = 4, *p < 0.05, **p < 0.01. Scale bar indicated by 50 µm.

To investigate whether this increase in *APLN* expression stems from ECs, we performed an RNA *in situ* hybridization for *APLN* RNA transcripts in combination with CD31 and CLDN5 IF staining, on the human prenatal lung explants treated with the different VEGF-A isoforms (Figures 5A–G; Supplementary Figure S3). VEGF-A121 alone was the only treatment to significantly increase the percentage of *APLN* + cells in the explants (VEGF-A121: 15.92 ± 1.694, n = 4, *p* = 0.0019) (Figure 5H). However, we observed a significant increase in the percentage of *APLN* + -CD31+-CLDN5+ cells in the explants treated with VEGF-A121, -145, -121-145, or -121-145–189 (Figure 5I). Those treated with VEGF-A165 or VEGF-A189 solely demonstrated an increasing trend (VEGF-A121: 12.30 ± 1.607, n = 4, *p =* 0.0291; VEGF-A145: 14.94 ± 1.620, n = 4, *p* = 0.0282; VEGF-A165: 11.80 ± 1.750, n = 4, *p* = 0.1715; VEGF-A189: 12.21 ± 2.400, n = 4, *p* = 0.3091; VEGF-A121-145: 19.25 ± 1.687, n = 4, *p* = 0.0289; VEGF-A121-145-189: 8.800 ± 0.6996, n = 4, *p* = 0.0267). These results indicate that the increase of *APLN* expression in the treated prenatal lung explants previously observed through RT-qPCR analysis, is most likely localized to the ECs.

## 4 Discussion

This work aimed to investigate the implication of VEGF-A and its different isoforms in CAP2 cells differentiation during human lung development. It was previously demonstrated in the mouse that Vegf-a188 increases the percentage of CAR4-positive ECs (Fidalgo et al., 2022) and we therefore hypothesized that VEGF-A isoforms also might be involved in human CAP2 differentiation.

The role of VEGF-A signaling in the developing human lung was first supported by a positive correlation between the expression of the various VEGF-A isoforms (*121, -145, -165, and 189*) and CAP2 markers (*APLN, EDNRB, HPGD, SOSTDC1*, and *TBX2)* in prenatal lung tissues aged from the late pseudoglandular to early canalicular stages (10–20 weeks of GA). There was simultaneous elevated expression of *VEGF-A121, -145,* and -*189* in conjunction with several CAP2 markers (*SOSTDC1, EDNRB, HPGD*), peaking around 18–20 weeks of GA. Interestingly, this stage of development corresponds to the establishment of the canalicular stage, which is when the alveolo-capillary barrier initiates (Yaremenko et al., 2024; Pereda et al., 2013). It is also noteworthy that VEGF-A121 expression increases during this developmental stage in the human lung, whereas previous studies have shown that the expression of its murine homolog, Vegf-a120, decreases during development (Ng et al., 2001). This suggests that lung capillary development could involve different mechanisms in mice and in humans. Therefore, despite the mouse model providing some insightful information, our data highlight the relevance of investigating this process in human.

Human CAP1 cells are considered progenitor-like, exhibiting a greater proliferative capacity than CAP2 (Vila et al., 2020). To examine if the different VEGF-A isoforms could play a role in EC proliferation, we then treated human prenatal lung explants with exogenous VEGF-A isoforms, alone or combined, and analyzed EC number and proliferation by IF staining. None of the treatments led to a significant increase in EC number or proliferation rate. On the contrary, explants treated with VEGF-A189 showed a reduction of CD31^+^ cells when compared to the untreated control. They also presented fewer CD31-Ki67 double-positive cells, meaning VEGF-A189 inhibits the proliferation of ECs. However, in a similar way, it was reported that mice fibrosarcoma cells that were modified to only express Vegf-a188 (homologous to human VEGF-A189) had lower proliferation than those expressing only Vegf-a120 or Vegf-a164 (Kanthou et al., 2014). These results imply that different isoforms of the same gene can have divergent, even opposing, biological effects. Moreover, we observed that ECs in the explants treated with VEGF-A189 displayed a more flattened morphology, reminiscent of differentiated ECs (Pereda et al., 2013). Indeed, it was demonstrated that ECs presented an elongated shape, suggesting vascular remodeling and the generation of distal angiogenesis in mice (Pereda et al., 2013). Although none of the isoforms increased EC proliferation, it is therefore possible that their role in prenatal lung capillary development may instead involve promoting EC differentiation or maturation.

Analysis of CAP2 markers in the treated explants indeed revealed that VEGF-A signaling likely contributes to the development and regulation of CAP2 cells. We observed that all the VEGF-A isoforms decreased *HPGD* expression, except VEGF-A189. Since the lungs are a major site of prostaglandin metabolism, pulmonary capillary cells, such as CAP2 cells, are known to exhibit high expression of 15-PGDH. This high expression makes it a specific marker for these cells (Piper et al., 1970). Our data seems to show that *HPGD* expression isn’t promoted by VEGF-A isoforms. In contrast, VEGF-A189 was the only isoform to significantly decrease *EDNRB* expression, while the others had no effect. Interestingly, endothelin, a peptide hormone that plays a role in the regulation of blood pressure and vasoconstriction, have been shown to promote the proliferation of microvascular ECs through activation of endothelin receptors, such as EDNRB (Morbidelli et al., 1995). Therefore, the observed anti-proliferative effect of VEGF-A189 could partially be explained by this downregulation of *EDNRB*. Additionally, and most interestingly, it was noted that all VEGF-A isoforms (except VEGF-A145) increased *APLN* expression. Apelin is a small peptide hormone that functions as an endogenous ligand for the G-protein-coupled apelin receptor (APLNR) and which plays role in cardiovascular homeostasis (Wa et al., 2024). It has been well documented that *APLN* is a tip cell-enriched gene and promotes a pro-angiogenic state in ECs (del Toro et al., 2010; Helker et al., 2020). Apelin is important in vasculature formation during embryonic development of various models (Cox et al., 2006; Kälin et al., 2007). Apelin also enhances cardiac neovascularization acting as a chemoattractant by recruiting the cKit+/Flk1+/Aplnr + progenitor cells during the early myocardial repair (Temp et al., 2012). FISH analysis of *APLN* transcripts in the endothelium of VEGF-A-treated explants suggests that the increased expression likely originates from ECs. Among all the isoforms, VEGF-A121 seems to induce the highest *APLN* expression in the human prenatal lung explants. Interestingly, VEGF-A121 is the only freely diffusible VEGF-A isoform, not binding neuropilin-1 or heparan sulfate, and is known to mostly play a role in early angiogenesis rather than vessel maturation (Peach et al., 2018; Kawamura et al., 2008; Nakatsu et al., 2003).

VEGF-A has been shown to activate several intra- and intercellular signaling pathways associated with survival, migration, proliferation, and differentiation (Shiying et al., 2017). Therefore, it would be interesting to perform scRNA-seq and determine how pathway analysis and cell communication within the prenatal human lung are influenced by the different VEGF-A isoforms. Increasing our understanding of VEGF-A isoforms could lead to the establishment of new therapeutic strategies. For instance, for refractory angina, a new drug (XC001) corresponding to an adenoviral vector expressing 3 of the VEGF-A isoforms (*VEGF-A121, -165, -189*) is currently under testing, after it has been shown that it increased local neo-angiogenesis in preclinical studies (Povsic et al., 2023).

Although we believe our study is highly relevant given its exclusive use of prenatal human samples, with findings that challenge recent murine studies, we acknowledge certain limitations that may impede a complete understanding of VEGF-A’s role in CAP2 development. Whereas our lung explants were cultured under normoxic conditions, we appreciate that *in utero* lung development takes place in a hypoxic environment. Studies have demonstrated that hypoxia is a major driver of parenchymal and vascular lung development by downregulating VEGF-A expression (Zhou et al., 2022; Kim and Byzova, 2014; Esquibies et al., 2008). Furthermore, others have shown that pulmonary endothelial heterogeneity and CAP2 differentiation increase after birth, when the concentration of oxygen is increased (Zepp et al., 2021). Therefore, although the explants used are excised from prenatal tissues, normoxic conditions may not fully recapitulate the vascular development driven by hypoxia. Therefore, future studies under hypoxic conditions would be valuable to assess the expression of the different VEGF-A isoforms, as well as evaluate its influence on capillary development. Another important limitation to acknowledge is the inherent variability within human samples, which may influence how individual samples respond to different treatments/chemical concentrations. To address this, we first established optimal concentrations via dose-response treatments on several samples, prior to initiating the study. Additionally, we ensured that each sample provided enough explants to complete a full experiment, allowing comparisons to be made across treatments of the same sample. This helped minimize variability. Finally, we do note that although several of our results showed statistically significant differences in gene expression, the fold change was often quite small, again likely stemming from the variability associated with human samples.

Taken together, our findings show that human VEGF-A189 plays a different role than its murine homolog Vegf-a188, which highlights differences between the mouse model and humans. Our results show that several VEGF-A isoforms increase the expression of the CAP2 marker *APLN* in human prenatal lung explants, suggesting an implication in early angiogenesis. However, since they did not induce the entirety of the CAP2 signature, it suggests that CAP2 differentiation is likely a multifactorial process, not solely dependent on VEGF-A signaling.

## Data Availability

The original contributions presented in the study are included in the article/Supplementary Material, further inquiries can be directed to the corresponding author.
