# Supplementary material for: VEGF-A isoforms induce the expression of APLN in endothelial cells during human prenatal lung development
Source: Front Cell Dev Biol. 2026 Jan 23;13:1729884. doi: 10.3389/fcell.2025.1729884 (PMC12876131; doi:10.3389/fcell.2025.1729884)
Supplement: Supplementary file 1 [file DataSheet1.pdf]

Supplementary Material

1 Supplementary Figures and Tables

1.1 Supplementary Figures

Supplemental Figure S1: experimental design of each step of the methodology. A- RNA extraction from native human prenatal lungs ranging from the late pseudoglandular (10 to 15 weeks of GA, n=24) to the early canalicular (16 to 20 weeks of GA, n=28) stages of development and CAP2 and VEGF-A marker gene expression analysis on the native human prenatal lung RNA. **B-** Fresh human prenatal lung explants from 16.3 to 21.6 weeks of GA (n=14) were treated with recombinant human VEGF-A (rhVEGF-A) isoforms either individually (VEGF-A121, VEGF-A145, VEGF-A165, VEGF-A189) or in defined combinations (VEGF-A121 + VEGF-A145, and VEGF-A121 + VEGF-A145 + VEGF-A189), with untreated explants serving as the negative control. Each treatment was refreshed every 24 hours over a 48-hour culture period. RNA extraction and gene expression analysis for the CAP2 marker expressions were performed. Tissue staining (H&E), and IF and FISH-IF staining were performed for respectively for CD31-Ki67 markers and CD31-CLDN5-*APLN* markers.

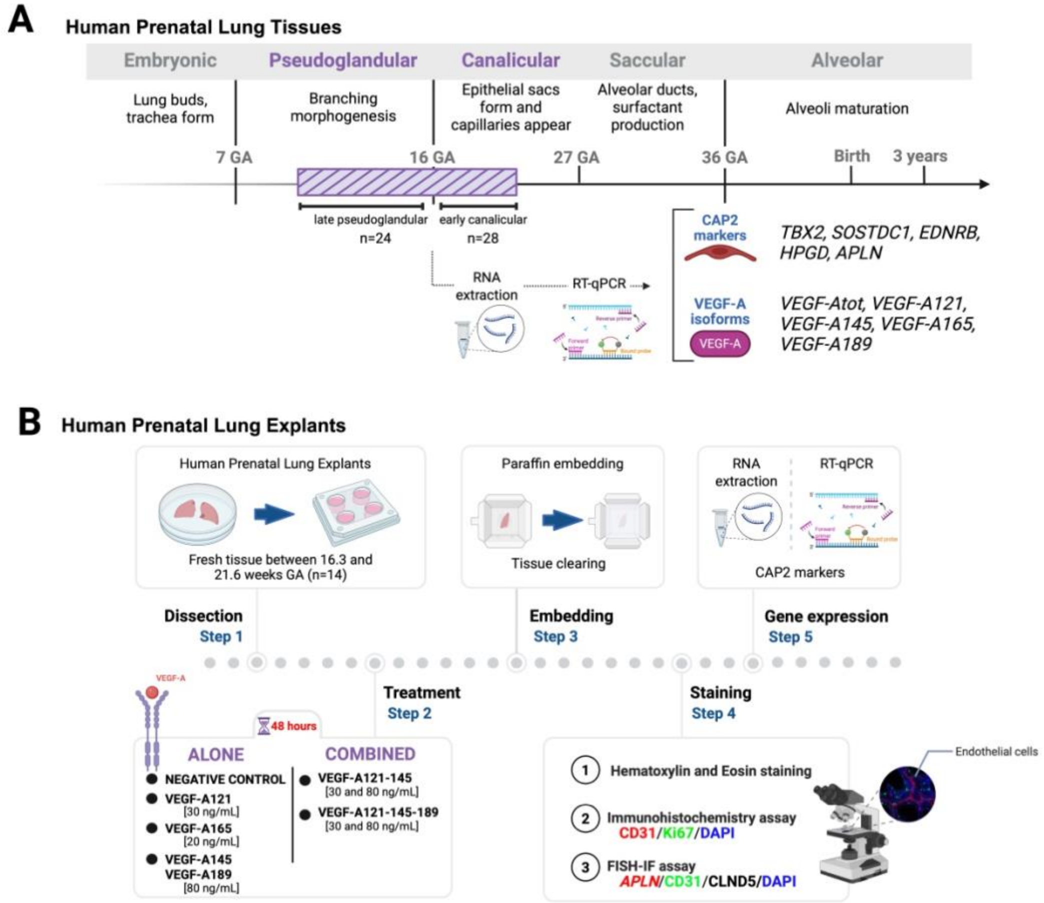

Supplemental Figure S2: Impact of VEGF-A isoforms on *SOSTDC1* and *TBX2* expression in human prenatal lung explants. RT-qPCR analysis of the expression of *SOSTDC1* and *TBX2* in human prenatal lung explants aged from the pseudoglandular to the canalicular stage (16 to 21 weeks of GA), untreated or treated with the different VEGF-A isoforms alone or in combinations (VEGF-A121, VEGF-A145, VEGF-A165, VEGF-A189, VEGF-A121-145, and VEGF-A121-145-189) for 48 hours. Results are shown as a dot plot with mean ± SEM, n=4-14.

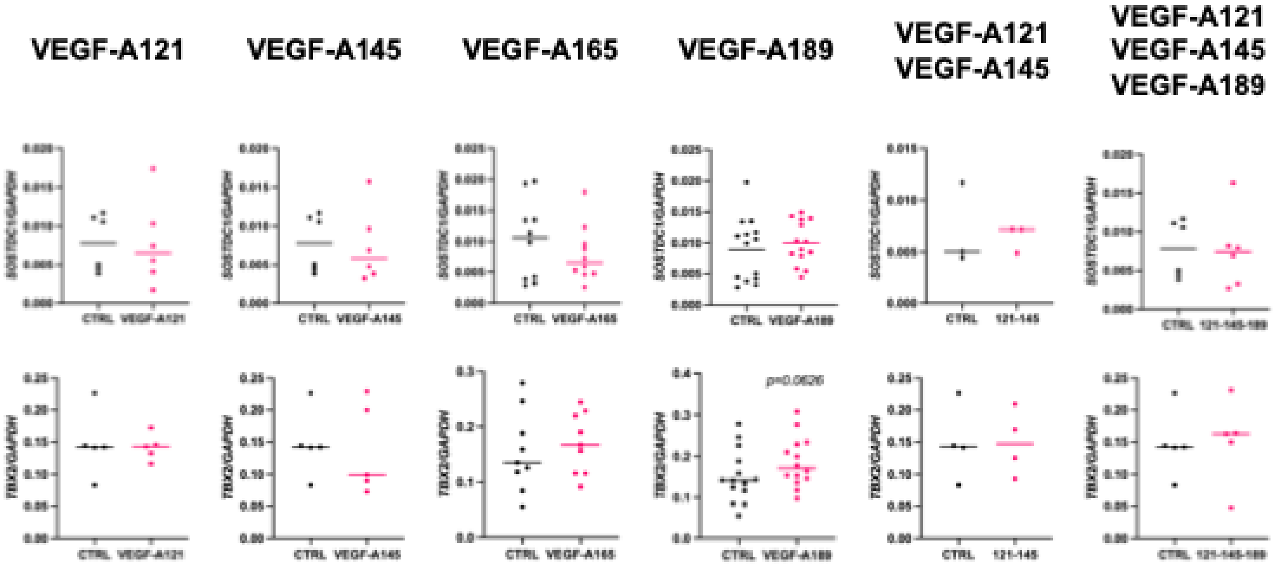

Supplemental Figure S3: Representative microscope images showing separated fluorescence channels and merged composites. Cy3 (red) corresponds to the RNA-FISH probe for *APLN*, GFP (green) marks CD31, Cy5 (far-red) labels CLAUDIN-5, and DAPI (blue) stains nuclei. For each treatment condition (VEGF-A121, VEGF-A145, VEGF-A165, VEGF-A189, VEGF-A121-145, and VEGF-A121-145-189), images are displayed as individual channels (Cy3, GFP, Cy5, DAPI) followed by the merged image. Scale bar = 50 µm.

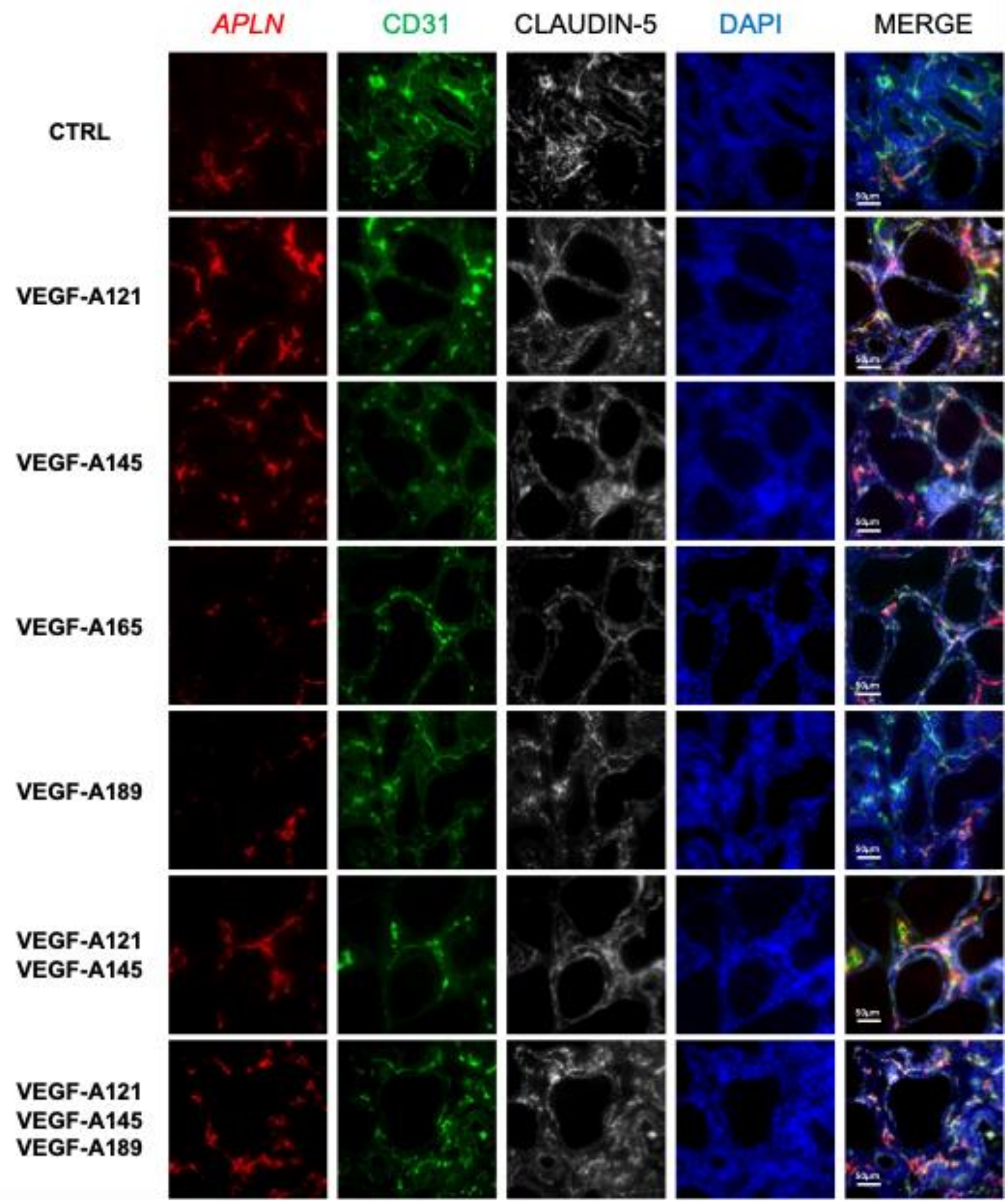

1.2 Supplementary Table

Supplemental Table S1: The human prenatal lung tissues used in this study for CAP2 marker and VEGF-A isoforms expressions

Sample Identifier	Sex	GA	Sample Identifier	Sex	GA	Sample Identifier	Sex	GA	Sample Identifier	Sex	GA
343	M	10.1	1430	F	12.4	282	F	16	605	M	18
439	UNK	10	357	F	14	375	F	16	627	M	18
957	UNK	10.1	419	M	14	378	F	16	1033	M	18.1
971	F	10	455	F	14.2	477	F	16	140	M	20
1077	M	10	497	M	14.5	737	F	16	154	M	20
1118	f	10.3	559	F	14.4	2306	M	16.2	221	M	20.3
1133	M	10.3	615	F	14	27640	F	16	292		20.1
1383	UNK	10.3	803	M	14.1	26	F	18.2	412	F	20.5
481	M	12.3	843	M	14	83	F	18	488	M	20.5
817	M	12.3	917	F	14.2	300	M	18	494	F	20.4
866	M	12	27632	F	14.7	328	M	18	602	M	20.2
963	M	12.5	62	F	16	550	M	18	2109	M	20.5
1008	M	12.6	65	M	16	584	F	18	27750	M	20.9
